# Parents’ Modeling During the COVID-19 Pandemic: Influences on Family Members’ Diet Quality and Satisfaction With-Food-Related Life in Dual-Earner Parents With Adolescent Children

**DOI:** 10.3389/fnut.2022.902103

**Published:** 2022-05-18

**Authors:** Berta Schnettler, Edgardo Miranda-Zapata, Ligia Orellana, Mahia Saracostti, Héctor Poblete, Germán Lobos, Cristian Adasme-Berríos, María Lapo, Katherine Beroiza, Klaus G. Grunert

**Affiliations:** ^1^Facultad de Ciencias Agropecuarias y Forestales, Universidad de La Frontera, Temuco, Chile; ^2^Scientific and Technological Bioresource Nucleus (BIOREN-UFRO), Universidad de La Frontera, Temuco, Chile; ^3^Centro de Excelencia en Psicología Económica y del Consumo, Núcleo de Ciencias Sociales, Universidad de La Frontera, Temuco, Chile; ^4^Universidad Católica de Santiago de Guayaquil, Guayaquil, Ecuador; ^5^Facultad de Educación, Centro de Investigación Escolar y Desarrollo (Cied-UCT), Universidad Católica de Temuco, Temuco, Chile; ^6^Universidad Autónoma de Chile, Temuco, Chile; ^7^Escuela de Trabajo Social, Universidad de Valparaíso, Valparaíso, Chile; ^8^Facultad de Economía y Negocios, Universidad de Talca, Talca, Chile; ^9^Departamento de Economía y Administración, Universidad Católica del Maule, Talca, Chile; ^10^MAPP Centre, Aarhus University, Aarhus, Denmark; ^11^University of Vaasa, Vaasa, Finland

**Keywords:** food parenting practices, modeling, diet quality, satisfaction with food-related life, dual-earner couples, adolescents

## Abstract

Reciprocal family influences in the food domain have been little explored, particularly during the COVID-19 pandemic. To fill in this gap, this study explored actor and partner effects between parents’ food modeling and parents’ and their adolescent children’s diet quality and satisfaction with food-related life (SWFoL); and the mediating role of diet quality between modeling and SWFoL. This study used a cross-sectional design. A sample of 430 different-sex dual-earner parents and one adolescent child were recruited in Rancagua, Chile, between March and June 2020. Parents answered the modeling dimension of the Comprehensive Feeding Practices Questionnaire. Parents and adolescents answered the Adapted Healthy Eating Index (AHEI) and the SWFoL Scale. Analyses were conducted using the Actor-Partner Interdependence Model and structural equation modeling. Results showed that one parent’s modeling enhanced diet quality for themselves, their partner, and the adolescents. Parents’ modeling was associated with their own SWFoL, directly and *via* their own diet quality. There were positive associations between mothers’ modeling and adolescents’ SWFoL; between mothers’ diet quality and fathers’ SWFoL; and between mothers’ modeling and fathers’ SWFoL *via* the fathers’ diet quality. Parents’ modeling can improve the three family members’ diet quality, while mothers’ modeling and diet quality showed to improve fathers’ and adolescents’ SWFoL.

## Introduction

Parents use an array of techniques or behaviors to influence what, when or how much their children eat. These techniques are known as food parenting practices (FPP) and they can be used by parents to promote healthy eating and prevent overweight and obesity in their children (1, 2). Parents remain responsible for feeding their children during adolescence (3, 4), but in this life stage adolescents seek autonomy in all life spheres, including the decisions regarding what and when to eat (5, 6). Adolescents change their dietary behaviors, engaging more with peers and eating outside the home (7, 8), and thus they decrease their involvement in family meals and possibly increase their consumption of convenience foods. These changes in adolescents’ eating habits have been found to contribute to a decline in diet quality and to an increased weight gain risk for adolescents (7, 9).

The COVID-19 pandemic has brought on changes in how parents apply FPP. Lockdown measures to reduce the risk of contagion enforced changes in behavioral patterns, including diet and eating habits (10, 11). Studies show that families get together more frequently for meals during the pandemic (12–15), which has entailed changes in how parents of young children exert FPP (11, 14). Nevertheless, there are scarce studies into FPP during the pandemic and their use on adolescents (11). Therefore, this study focuses on the effects of parents’ modeling of healthy food choices on their adolescent children. Modeling is one of the multiple FPP in which parents can engage in Jennings et al. (16), and it plays a vital role in shaping children’s food preferences (3, 5, 17). Using modeling to promote healthy eating involves an active demonstration from parents to children (6, 18). Parents thus become a point of reference on eating behaviors and can influence children’s long-term food choices and eating habits (19). The literature has reported positive relationships between parents’ modeling of healthy food choices and diet outcomes in adolescents (5, 9, 10, 20–24). These studies, however, have focused on samples from developed countries (23, 25, 26), and on mother-child dyads (17, 23, 25–29). Father-child dyads have been less explored in terms of FPP. Evidence to date suggests that fathers are becoming more involved in childcare tasks such as feeding, partly due to the increase of dual-earner families (15, 19, 28, 30, 31), and more markedly due to changes in work-home dynamics in response to the COVID-19 pandemic (13, 32).

Another gap in knowledge relates to the influence of parents’ FPP and family dietary behaviors. FPP may reflect parents’ eating habits (25, 29, 33–35), but it is not clear whether and how parents’ dietary behaviors are linked to the FPP exerted on adolescents (29). Most of the literature has also assumed a unidirectional relationship between FPP and children’s dietary behaviors, omitting the exploration of reciprocal influences between parents and children (25, 35–37). Addressing the reciprocity between parents and children’s eating behaviors can help identify children’s eating and weight development processes (25). This knowledge can inform overweight prevention and intervention programs, particularly in contexts such as Chile, where 60% of children, and three out of four adults, are overweight or obese (38).

In samples of adolescents and adults, FPP aimed to implement healthy eating habits have been linked to higher levels of emotional wellbeing and satisfaction in the food domain (4, 39–43). The latter construct, known as satisfaction with food-related life [SWFoL, (44)], defines a person’s overall cognitive assessment of their food and eating habits, covering from meal planning, shopping and meal preparation, consumption, and disposal. To the best of the authors’ knowledge, SWFoL has not been explored in its relationships with parental modeling and diet quality at a family level. This exploration becomes relevant, particularly in dual-earner parents, because high job demands have been associated with lower diet quality, not only for the worker but also for their families, given that personal resources such as time and energy are invested in workplace responsibilities instead of on food-related tasks [e.g., (20, 45, 46)].

**FIGURE 1:**
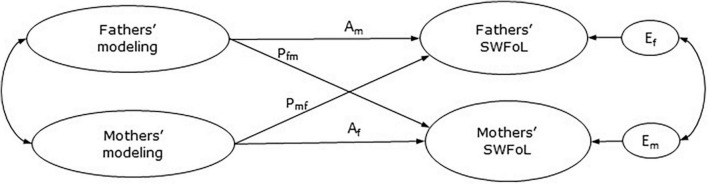
Basic actor-partner interdependence model of modeling and satisfaction with food-related life (SWFoL). A_m_, actor effect of father’s modeling on his own SWFoL; A_f_, actor effect of mother’s modeling on her own SWFoL; P_fm,_ partner effect of father’s modeling on mother’s SWFoL; P_mf,_ partner effect of mothers’ modeling on fathers’ SWFoL; E_f_ and E_m,_ residual errors on SWFoL for the father and mother, respectively.

Against this background, this study examined the influence of both parents’ modeling on their own diet quality and SWFoL, and on their adolescent children’s diet quality and SWFoL. This study was conducted in different-sex dual-earner parents with adolescent children in a Latin American country during the COVID-19 pandemic. Data was analyzed using the Actor-Partner Interdependence Model [APIM, (47)] (Figure 1). Therefore, the aims of this study were to explore the actor and partner effects between the parents’ modeling and mothers’, fathers’, and adolescents’ diet quality and SWFoL; and to explore whether diet quality have a mediating role between both parents’ modeling and the three family members’ SWFoL.

### Relationships Between Parental Modeling, Diet Quality and Satisfaction With Food-Related Life

Studies regarding the impact of parental modeling on adolescent children’s outcomes are scarce (23), but previous findings have shown links between modeling of healthy food choices and children’s healthy dietary intake (16, 23). Moreover, it has been reported that parents’ food intake strongly predicts that of their children (26). For instance, studies in the European Union and the United States have shown that parental modeling of healthy food choices is positively associated with dietary outcomes in adolescents such as healthier diet (24, 48), greater intake of fruits and vegetables (8, 21, 27, 48, 49), and fewer consumption of sugar-sweetened beverages and palatable snack foods (5, 22, 27, 49).

Nevertheless, FPP may not only be related to children’s eating habits, but to the eating habits of other family members (20). Evidence from the United States and European countries indicate a high correlation between parents’ and adolescents’ dietary behaviors (33, 34), while parents’ FPP are consistent not only with their adolescent children’s dietary behavior, but their own diet (29). In different-sex couples, the literature also shows that spouses can influence one another on their eating behaviors (50). Findings in this line show that women are more influenced by their male partner than vice versa, and this distinction has been explained by women’s traditional gender socialization regarding a higher sensitivity toward their partners than men (51, 52). Nevertheless, men’s eating behaviors can also be influenced by their female partners (53). On this basis, we propose that parents’ modeling can influence not only their adolescent children’s diet quality, but also their own and their partner’s, in the following hypotheses:

H1. Parents’ modeling of healthy food choices is positively associated with their own diet quality (actor effects).

H2. Modeling of healthy food choices of one parent is positively associated with the diet quality of (a) the other parent and (b) of the adolescent (partner effects).

Healthier eating habits have been positively associated with higher levels of SWFoL in individual-level research in adults [e.g., (36, 39, 40, 54–57)] and adolescents (39, 57, 58). At the same time, findings show that eating behaviors and SWFoL are correlated among family members (28, 29, 34, 36), and that parents’ SWFoL is linked to healthy eating behaviors in the family (36, 59). Therefore, it can be expected that parental modeling and associated dietary outcomes are linked to both adolescents’ and parents’ SWFoL, as proposed in the following hypotheses:

H3. Diet quality is positively associated with satisfaction with food-related life for (a) fathers, (b) mothers, and (c) adolescents (actor effects).

H4. Diet quality of one parent is positively associated with satisfaction with food-related life of (a) the other parent, and (b) the adolescent (partner effects).

H5. Diet quality of adolescents is positively associated with their parents’ satisfaction with food-related life (partner effects).

Outcomes of different FPP on adolescents’ and their parents’ wellbeing have been scarcely studied, but research to date shows that structure-related and autonomy supporting FPP can benefit both parents’ and children’s wellbeing (9, 41). This beneficial relationship is thought to occur because these FPP are based on a supportive parental approach to healthy eating that also accounts for the child’s emotional and psychological needs (2, 25). Studies on monitoring and SWFoL and life satisfaction [i.e., the individual’s assessment of their overall life conditions or specific domains, (60)] also support these findings. Some studies have found that maternal monitoring of child snacking behavior is linked to both mothers’ and adolescents’ SWFoL (39, 41). Other studies have reported that structured meals, a healthy food-home environment, and lack of pressure to eat increase emotional wellbeing and life satisfaction in adolescent’s and their parents (40, 43), and these relations continue during the COVID-19 pandemic in young adults (12). Therefore, we propose that modeling has a beneficial influence on parents’ and adolescents’ SWFoL, with crossover effects of modeling and SWFoL between both parents:

H6. Modeling of healthy food choices is positively associated with satisfaction with food-related life for each parent (actor effects).

H7. Modeling of healthy food choices of one parent is positively associated with satisfaction with food-related life of (a) the other parent, and (b) of the adolescent (partner effects).

**FIGURE 2:**
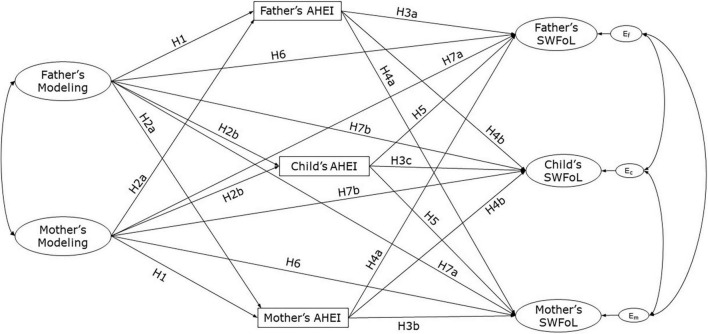
Conceptual model of the proposed actor and partner effects of both parent’s modeling on the three family members’ diet quality (measured by the Adapted Healthy Eating Index, AHEI) and Satisfaction with Food-related Life (SWFoL) in dual-earner parents with adolescent children. Ef, Ec, and Em, residual errors on SWFoL for the fathers, mothers and their adolescent children, respectively. The indirect effects of AHEI (H8) were not shown in the conceptual path diagram to avoid cluttering the figure.

Figure 2 displays the conceptual model including the first seven hypotheses. The effects proposed in H6 and H7 imply that modeling of healthy food choices could lead to more SWFoL independently of the effect of the modeling on diet quality. To the best of our knowledge, there are no published studies exploring the mediating role of diet quality between FPP and SWFoL. Previous studies suggest mechanisms supporting positive relationships between parental modeling and healthy dietary intake in parents (29, 33, 34) and children (8, 21, 27, 48, 49); between healthier eating habits and higher levels of SWFoL in samples of adults [e.g., (39, 50–57)] and adolescents (39, 57, 58); and between parents’ SWFoL and their family members’ healthy eating behaviors (36, 59, 61). On this base, we posed the last hypothesis of this study:

H8. Diet quality has a mediating role between both parents’ modeling of healthy food choices and satisfaction with food-related life for the three family members (actor and partner effects).

One last consideration for this proposal derives from the Latin American context in which this study is conducted. In this cultural context, fathers tend to engage less in FPP than mothers, because the latter are traditionally tasked with food purchase and meal preparation (20, 28, 33, 34, 40). However, other research with Latino families suggests that fathers can also improve their children’s eating behaviors by exerting FPP, buying and preparing foods, and participating in mealtimes (27, 34, 62). Another study reported gender differences in FPP and established the differential roles that mothers and fathers play in the development of their children’s eating habits (28). Thus, we expect that the proposed associations between parental modeling, diet quality and SWFoL may show different patterns based on the parent’s gender.

## Materials and Methods

### Sample and Procedure

This study used a cross-sectional design. The sample was composed of 430 dual-earner families including mother, father (married or cohabiting) and one adolescent child ages 10–16 (Table 1). This study is part of a wider research on the relations between work, family, and food-related life in Chilean families. Sample size was determined considering 10 participants for each item of each scale used in this project. Families were recruited using non-probability sampling in Rancagua, Chile. Potential participants were contacted *via* seven schools that serve diverse socioeconomic backgrounds in the city. School principals signed authorization letters to conduct the research with students from fifth grade of primary level (minimum age of 10 years) to the first grade of secondary level (maximum age of 16 years). Parents of students in these grades received information from trained interviewers about the study’s objectives, the structure of the questionnaire, and the guarantees of anonymity and confidentiality of the responses.

**TABLE 1:** Sample characteristics (*n* = 430).

Characteristic	Total sample	*P*-value^a^
**Age [Mean (*SD*)]^a^**		
Mother	39.5 (6.6)	<0.001
Father	42.3 (7.8)	
Adolescent	13.0 (2.0)	
**Adolescents’ gender [%, (*n*)]**		
Male	46.3 (199)	
Female	53.7 (231)	
Number of family members [Mean (*SD*)]	4.3 (1.0)	
Number of children [Mean (*SD*)]	2.2 (0.8)	
**Socioeconomic status [%, (*n*)]**		
High	3.7 (57)	
Middle	83.0 (357)	
Low	3.7 (16)	
**Number of days/week families ate together [Mean (*SD*)]**		
Breakfast	3.5 (2.7)	
Lunch	4.9 (2.4)	
Supper	6.0 (1.9)	
Dinner	2.2 (3.1)	
**Number of days/week families eat different types of foods [Mean (*SD*)]**		
Homemade foods	6.4 (1.3)	
Buy ready-to eat food	0.4 (1.2)	
Order food at home	0.6 (0.7)	
Eat at restaurants	0.2 (0.5)	
Eat at fast-food outlets	0.3 (0.6)	
**Number of hours per day spent cooking during the week [Mean (*SD*)]^b^**		
Mother	2.6 (1.3)	<0.001
Father	1.2 (1.3)	
Another person	0.9 (1.5)	
**Number of hours per day spent cooking on the weekend [Mean (*SD*)]^b^**		
Mother	3.1 (1.6)	<0.001
Father	1.7 (1.4)	
Another person	0.7 (1.2)	
Type of employment [%, (*n*)]^c^		<0.001
Woman employee	62.8 (270)	
Woman self-employed	37.2 (160)	
Man employee	75.3 (324)	
Man self-employed	24.7 (106)	
**Working hours [%, (*n*)]^c^**		
Woman working 45 h per week	44.0 (189)	<0.001
Woman less than 45 h per week	56.0 (241)	
Man working 45 h per week	67.2 (289)	
Man working less than 45 h per week	32.8 (141)	

*^a^Independent sample t-test.*

*^b^Analysis of variance.*

*^c^P-value corresponds to the (bilateral) asymptotic significance obtained in Pearson’s Chi-square Test.*

Families who agreed to participate in the study provided one e-mail address to receive the links to three surveys, one for each family member, between March and July 2020. Trained interviewers maintained contact with the families by phone, to answer questions about the study and to monitor the responses to the questionnaire. The COVID-19 pandemic was declared by mid-March 2020 in Chile, and the city of Rancagua was on mandatory lockdown during June and July 2020. However, most workers in the country, particularly women, began to work from home at the start of the pandemic (32).

For mothers and fathers, the first page of the questionnaire showed the informed consent form, while for adolescents it showed an informed assent form. The three family members were asked to click a box to confirm their agreement to participate before starting the questionnaire. All responses were recorded in the QuestionPro platform (QuestionPro Inc.) in separate databases for each family member. When the three family members submitted their responses, the family received a bank transfer for 15 USD as retribution for their participation.

A pilot test for this study was conducted with fifty families in Temuco, Chile. The recruitment method and data collection procedure were the same as the ones declared above. Results of this pilot test were satisfactory, with no changes required to the method nor the questionnaire. This study has been approved by The Ethics Committee of the Universidad de La Frontera.

### Measures

The following scale was answered only by mothers and fathers:

#### Modeling of Healthy Food Choices

Four items were adapted from the modeling factor of the *Comprehensive Feeding Practices Questionnaire* (CFPQ). The CFPQ (18) is a 12-factor questionnaire that measures 12 FPP, including the modeling dimension. Mothers and fathers answered the validated adapted version of the modeling factor (6) for adolescents’ parents, which measures that “parents actively demonstrate healthy eating for the child” (i.e., 1. “I model healthy eating for my child by eating healthy myself,” 2. “I try to eat healthy foods in front of my child, even if they are not my favorite,” 3. “I try to show enthusiasm about healthy foods,” 4. “I show my child how much I enjoy eating healthy foods”). Melbye and Hansen (63) reported a Cronbach’s alpha of 0.66 for the modeling dimension of the adapted CFPQ in a sample of parents of adolescents in Norway. In this study, the Spanish adapted version of this measure was used (64). Participants answered to each item using a 5-point Likert scale (1: disagree, 5: agree). Modeling scores were obtained by summing the scores from the four items, with higher scores representing higher modeling in parents.

The three family members answered the following instruments:

#### Adapted Healthy Eating Index

This instrument measures diet quality and is an adaption of the US-HEI (65) into Spanish developed by Norte and Ortiz (66). Participants are asked to report the frequency of consumption of nine food groups: 1. Cereal and derivatives; 2. Vegetables; 3. Fruit; 4. Milk and dairy products; 5. Meats; 6. Legumes; 7. Sausages and cold meats; 8. Sweets, 9. Soft drinks with sugar. The frequency for each food group is converted to a score from 0 to 10, as proposed by Norte and Ortiz (66) following the degree of compliance with food daily and weekly recommendations. For the first nine variables, respondents indicated their consumption frequency of the target food. Each variable received a score, ranging from 0 to 10, according to the degree of compliance with dietary recommendations [criteria is available in reference (66)]. The last variable, relating to diet variety, is constructed using the consumption frequency of the nine target foods: two points were received if the respondent complied with each of the daily recommendations and one point was received if he/she complied with each of the weekly recommendations. The overall Adapted Healthy Eating Index (AHEI) score was calculated by adding the scores obtained in each of the variables. The maximum possible score is of 100 points. Scores above 80 points indicate a “healthy” diet; scores between 51 and 80 points suggest that the diet “requires changes”; and scores below 50 points indicate “unhealthy” diets (65).

#### Satisfaction With Food-Related Life

The SWFoL (44) is a one-dimension, five-item scale that measures an individual’s overall assessment of their food and eating habits (e.g., “Food and meals are very positive elements in your life”). The Spanish version of the SWFoL was used (67), which has been validated and it has shown good internal consistency in samples of adults, adolescents and dual-earner parents in Chile [e.g., (4, 15, 32, 36, 39–41, 57, 61)]. Respondents indicate their degree of agreement with each statement using a 6-point Likert scale (1: completely disagree; 6: completely agree). SWFoL scores are obtained by summing the scores from the five items, and higher scores indicate higher SWFoL.

The three family members reported their age; adolescents reported their gender. Parents answered questions about their type of employment, the number of working hours per week and their monthly income. Mothers reported the number of family members, the number of children, the number of days per week that all family members eat together (breakfast, lunch, supper, and dinner); the number of days per week that they consumed homemade food, buy ready-to-eat food, order food at home, or eat at restaurants or fast-food outlets; and the number of hours per day that they, their male partner and other person spent cooking during the week and on weekends. They were asked their own approximate weight and height as well as from the fathers and the children to determine body mass index (BMI) in kg/m^2^. The family socioeconomic status (SES) was determined based on the total household income and its size. The definition of SES in Chile considers two variables: total household income and household size. Total household income is the fundamental variable for socioeconomic segmentation, due to its predictive power on access to goods and services, and because the inverse relationship is much weaker: access to goods and services is not a good predictor of income. The size of the household exerts a restriction on purchasing power: When an additional member is added to the household without increasing income, basic expenses increase albeit in a sub-proportionate way considering economies of scale. The combination of ranges of the household monthly income and the number of family members in a matrix determines the SES (68).

### Data Analysis

Descriptive analyses were conducted using the Statistical Package for Social Sciences (SPSS) version 23.0 (IBM Corp. Released 2015. IBM SPSS Statistics for Windows, Armonk, NY: IBM Corp). The actor-partner interdependence model (APIM) with distinguishable dyads was tested using structural equation modeling (SEM) (47) with Mplus 8.4. In this study, actor effects are those outcomes predicted by the individuals’ own characteristics, while partner effects are outcomes from one member of the dyad predicted by the characteristics of the other member. Members of a dyad are both an actor and a partner in the analysis. In this study, dyads are composed of mother-father, and parent-adolescent. The actor and partner effects tested were modeling from mothers and fathers, and the three family members’ diet quality and SWFoL.

In the APIM, other effects were controlled for. First, the influence of modeling from one parent to the other was controlled for by specifying a correlation between this variable reported by each parent. Other sources of interdependence between individuals were controlled for following guidelines by Kenny et al. (47), by specifying correlations between the residual errors of the dependent variable (SWFoL) of the three family members. Other effects that were controlled for were those of family SES, number of children, parents’ and adolescents’ age, both parents’ number of working hours and type of employment, and family supper times per week. These variables with direct effects on the dependent variables of the three family members (diet quality and SWFoL) were thus incorporated in the model.

To conduct the SEM, structural model parameters were estimated using weighted least square mean and variance adjusted (WLSMV). Because the items were on an ordinal scale, the polychoric correlation matrix was used. A good model fit of the data was determined with the following values: when values are above 0.95 for the Tucker-Lewis index (TLI) and the comparative fit index (CFI); and when values are below 0.06 for the root mean square error of approximation [RMSEA, (69)]. Lastly, to test the mediating role of diet quality, a SEM through a bias-corrected (BC) bootstrap confidence interval using 1,000 samples (70) was conducted. A mediating role is found when BC confidence intervals do not include zero.

## Results

### Sample Description

Table 1 shows the sociodemographic characteristics of the sample composed by 430 mother-father-adolescent families. The average age for mothers was 39.5 years old, for fathers 42.3 years, and for adolescents 13.0 years. The difference between mothers’ and fathers’ age was significant (*p* < 0.001). In the adolescent subsample, 53.7% were female. On average, families had four members and two children, and most families belonged to a middle SES.

Reports about frequency of family meals showed that families got together for breakfast, lunch, and supper more than 3 days per week, and homemade food was consumed frequently. The main responsibility for food decision-making and purchases in the household was most often shared by both parents, followed by mothers having main responsibility. Mothers spent significantly more hours per day cooking during the week and on weekends, compared to fathers and other persons (*p* < 0.001), and fathers spent more hours in this same activity than other persons. “Other persons” referred most frequently to grandmothers, adult children, and domestic service. Most mothers and fathers were employees as opposed to independent workers. A greater proportion of fathers, compared to mothers, worked full time (45 h per week in Chile, *p* < 0.001) and were employees (*p* < 0.001).

In terms of body mass index, following the norms of the World Health Organization (WHO), 28.4% of mothers had a body mass index in the normal range (BMI: 18.5–24.9), 44.2% were overweight (BMI: 25.0–29.9) and 27.9% were obese (BMI ≥ 30). In fathers, 16.3% had a body mass index in the normal range, 57.0% were overweight, and 26.7% were obese. For adolescents, the guidelines used are those from the WHO (71) and the Technical Norm of Nutritional Evaluation of children from 5 to 19 years old of the Ministry of Health of Chile (72). According to these guidelines, 0.5% of adolescents in this sample had a body mass index that denote thinness (≤ −1 to −1.9 *SD*), 27.9% were in the normal range (+0.9 to −0.9 *SD*), 44.2% were overweight (≥+1 to + 1.9 *SD*), and 27.4% obese (≥ + 2 *SD*).

Table 2 shows the average score and the correlations for both parents’ modeling, diet quality (measured by the AHEI) and SWFoL. All of the correlations were significant and in the expected directions. Mothers scored significantly higher than fathers in modeling (*t* = 4.421, *p* < 0.001). Fathers scored significantly lower than mothers and their adolescent children in the AHEI (*F* = 12.524, *p* ≥ 0.001), whereas mothers and adolescents did not differ from one another. However, according to the cut-off point proposed by Kennedy et al. (65), the three family members had AHEI average scores indicating that their diet “requires changes.” Adolescents scored significantly higher than their mothers and fathers in the SWFoL (*F* = 18.321 *p* < 0.001), while fathers scored significantly higher than mothers.

**TABLE 2:** Descriptive statistics and correlations for both parent’s modeling and the three family members’ diet quality (measured by the Adapted Healthy Eating Index, AHEI) and Satisfaction with Food-related Life (SWFoL) in dual-earner parents with adolescent children (*n* = 430).

	*M* (*SD*)	Correlations							
		1	2	3	4	5	6	7	8
1. Mother’s modeling	16.32 (3.20)	−	0.328***	0.392***	0.210***	0.236***	0.267***	0.193***	0.199***
2. Father’s modeling	15.25 (2.91)		1	0.208***	0.311***	0.152***	0.129**	0.358***	0.177***
3. Mother’s AHEI	65.07 (12.52)			1	0.500***	0.593***	0.309***	0.124**	0.220***
4. Father’s AHEI	60.89 (14.10)				1	0.508***	0.146**	0.303***	0.125**
5. Adolescent’s AHEI	64.78 (14.36)					1	0.124**	0.189***	0.164**
6. Mother’s SWFoL	22.13 (4.52)						1	0.303***	0.298***
7. Father’s SWFoL	23.13 (4.30)							1	0.351***
8. Adolescent’s SWFoL	23.94 (4.35)								1

**p < 0.01, ***p < 0.001.

### Actor-Partner Interdependence Model Results: Testing Actor-Partner Hypotheses

In this study, the standardized factor loadings of the modeling factor ranged from0.732 to 0.933 for mothers and from 0.818 to 0.909 for fathers, all statistically significant (*p* < 0.001). The Average Variance Extracted (AVE) values were higher than 0.50 (AVE mothers = 0.71, fathers = 0.76). The modeling factor showed good internal reliability, as the Omega coefficient was 0.91 for mothers and 0.93 for fathers. The standardized factor loadings of the SWFoL scale were all statistically significant (*p* < 0.001), and ranged from 0.681 to 0.930 for mothers, from 0.597 to 0.931 for fathers, and from 0.655 to 0.847 for adolescents. The AVE values were higher than 0.50 (AVE mothers = 0.63, fathers = 0.63, adolescents = 0.58). The SWFoL scale showed good internal reliability, as the Omega coefficient was 0.89 for mothers, 0.89 for fathers, and 0.87 for adolescents.

**FIGURE 3:**
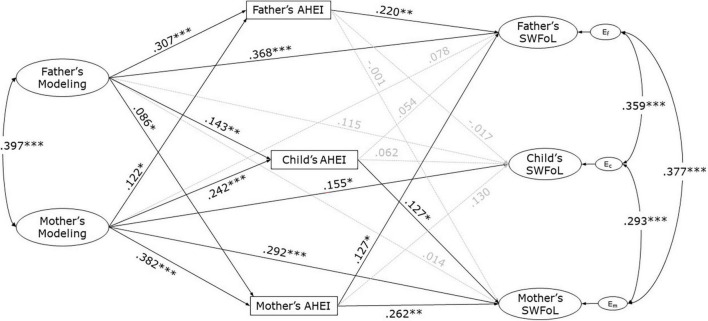
Actor-partner interdependence model of the effect of both parents’ modeling on the three family members’ diet quality (measured by the Adapted Healthy Eating Index, AHEI) and Satisfaction with Food-related Life (SWFoL) in dual-earner parents with adolescent children. E_f_, E_c_ and E_m,_ residual errors on SWFoL for fathers, mothers and their adolescent children, respectively. *p < 0.05, ^**^p < 0.01, ^***^p < 0.001. The control for the effects of family SES, number of children, parents’ and adolescents’ age, both parents’ number of working hours and type of employment, and times per week in which the family members had supper together on the dependent variables of the three family members (AHEI and SWFoL) were not shown in the path diagram to avoid cluttering the figure.

The results from the estimation of the structural model are shown in Figure 3. The model that assessed the APIM associations between the mothers’ and fathers’ modeling and the three family members’ AHEI and SWFoL had a good fit with the data (CFI = 0.983; TLI = 0.978; RMSEA = 0.036). A significant correlation (covariance) was found between modeling of both parents (*r* = 0.397, *p* < 0.001). Significant correlations were also found between the residual errors of mothers’ and fathers’ SWFoL (*r* = 0.377, *p* < 0.001), between mothers’ and adolescents’ SWFoL (*r* = 0.293, *p* < 0.001), as well as between fathers’ and adolescents’ SWFoL (*r* = 0.359, *p* < 0.001).

H1 tested actor effects, stating that parents’ modeling of healthy food choices is positively associated with their own diet quality. The path coefficients (standardized) indicate that fathers’ (γ = 0.307, *p* < 0.001) and mothers’ (γ = 0.382, *p* < 0.001) modeling was positively associated with their own AHEI. These findings supported H1 for both parents.

H2 tested partner effects, stating that modeling of healthy food choices of one parent is positively associated with the diet quality of the other parent (H2a) and of the adolescent (H2b). Results showed that fathers’ modeling was positively associated with mothers’ AHEI (γ = 0.086, *p* = 0.044), and that mothers’ modeling was positively associated with fathers’ AHEI (γ = 0.122, *p* = 0.010). Likewise, fathers’ (γ = 0.143, *p* = 0.003) and mothers’ (γ = 0.242, *p* < 0.001) modeling was positively associated with adolescents’ AHEI. These findings supported H2a and H2b.

H3 tested actor effects for the three family members, that is, diet quality is positively associated with SWFoL for fathers (H3a), mothers (H3b), and adolescents (H3c). The results indicate that fathers’ (γ = 0.220 *p* = 0.018) and mothers’ (γ = 0.262, *p* = 0.001) AHEI was positively associated with their own SWFoL. By contrast, adolescents’ AHEI was not significantly associated with their own SWFoL (γ = 0.062, *p* = 0.297). These findings supported H3 for both parents (H3a and H3b) but not for adolescents (H3c).

H4 tested partner effects, stating that the diet quality of one parent is positively associated with the SWFoL of the other parent (H4a) and of the adolescent (H4b). Fathers’ AHEI was not significantly associated with mothers’ SWFoL (γ = −0.001, *p* = 0.990), nor with their adolescent children’s SWFoL (γ = −0.017, *p* = 0.768). The mother’s AHEI was positively associated with the father’s (γ = 0.127, *p* = 0.049), but not with their adolescent children’s (γ = 0.130, *p* = 0.051) SWFoL. These findings partially supported H4a, while they did not support H4b.

H5 stated that adolescents’ diet quality is positively associated with their parents’ satisfaction with food-related life. The adolescents’ diet quality was not significantly associated with the fathers’ SWFoL (γ = 0.054, *p* = 0.326), but it was positively associated with their mothers’ SWFoL (γ = 0.127, *p* = 0.040), and thus H5 was partially supported.

H6 stated that modeling of healthy food choices is positively associated with satisfaction with food-related life for each parent. This relationship can be direct and/or mediated by diet quality. As shown in Figure 3, the path coefficients indicate that fathers’ (γ = 0.368, *p* < 0.001) and mothers’ (γ = 0.292, *p* < 0.001) modeling was directly and positively associated with their own SWFoL. These findings supported H6 for both parents even when not taking the possible mediation effect into account.

H7 examined partner effects, stating that modeling of healthy food choices of one parent is positively associated with the SWFoL of the other parent (H7a) and of the adolescent (H7b). There was no significant direct link between fathers’ modeling and mothers’ SWFoL (γ = 0.014, *p* = 0.798). Likewise, there was no significant direct link between mothers’ modeling and fathers’ SWFoL (γ = 0.078, *p* = 0.193). While fathers’ modeling (γ = 0.115, *p* = 0.064) was not directly associated with the adolescents’ SWFoL, mothers’ modeling was positively and directly associated with the adolescents’ SWFoL (γ = 0.155, *p* = 0.015). Before establishing a conclusion about H7, however, indirect effects must be explored in which the relationship between modeling and SWFoL is mediated by diet quality (H8).

Most of the control variables did not affect the model significantly (see Supplementary Information). The family SES positively affected the mothers’ (γ = 0.170, *p* < 0.01) and the adolescents’ (γ = 0.128, *p* < 0.05) AHEI as well as the mothers’ SWFoL (γ = 0.134, *p* < 0.01). Namely, those mothers belonging to the high SES had a higher score on the AHEI and experienced higher levels of SWFoL than those of lower SES, while adolescents belonging to the high SES had a higher score on the AHEI than those of lower SES. The mothers’ type of employment positively affected her own (γ = 0.116, *p* < 0.05) and their adolescent children’s AHEI (γ = 0.118, *p* < 0.05), meaning that self-employed mothers and their adolescent children had higher AHEI scores than employed mothers and their adolescent children. The number of family supper times per week positively affected the adolescents’ AHEI (γ = 0.106, *p* < 0.05) and the fathers’ SWFoL (γ = 0.110, *p* < 0.05).

### Testing Mediating Roles of Diet Quality

The last hypothesis of this study proposed the mediating role of the three family members’ diet quality between both parents’ modeling of healthy food choices and the three family members’ satisfaction with food related life (H8). The role of the mother’s diet quality (i.e., AHEI score) as a mediator in the relationship between her own modeling and SWFoL was supported by a significant indirect effect obtained with the bootstrapping confidence interval procedure (standardized indirect effect = 0.062, 95% CI = 0.028, 0.097), as the confidence intervals did not include zero (Table 3). Similarly, the role of the father’s diet quality as a mediator in the relationship between his own modeling and SWFoL was supported by a significant indirect effect (standardized indirect effect = 0.034, 95% CI = 0.016, 0.053). In addition, the role of the father’s diet quality as a mediator in the relationship between the mother’s modeling and the father’s SWFoL was supported by a significant indirect effect (standardized indirect effect = 0.016, 95% CI = 0.001, 0.031). No other indirect effects of diet quality were found, as the confidence intervals did include zero (Table 3).

**TABLE 3:** Bias-corrected confidence intervals of specific mediation effects of the three family members’ diet quality (measured by the Adapted Healthy Eating Index, AHEI).

Specific indirect effects	Estimate	Lower 2.5%	Upper 2.5%	*P*-value
Mothers’ modeling → Mothers’ AHEI → Mothers’ SWFoL	0.062	0.028	0.097	<0.001**
Mothers’ modeling → Fathers’ AHEI → Mothers’ SWFoL	0.000	−0.008	0.008	0.990
Mothers’ modeling → Adolescents’ AHEI → Mothers’ SWFoL	0.019	−0.002	0.040	0.073
Fathers’ modeling → Mothers’ AHEI → Mothers’ SWFoL	0.012	−0.001	0.025	0.073
Fathers’ modeling → Fathers’ AHEI → Mothers’ SWFoL	0.000	−0.017	0.017	0.990
Fathers’ modeling → Adolescents’ AHEI → Mothers’ SWFoL	0.009	−0.001	0.020	0.079
Mothers’ modeling → Mothers’ AHEI → Fathers’ SWFoL	0.030	−0.001	0.060	0.054
Mothers’ modeling → Fathers’ AHEI → Fathers’ SWFoL	0.016	0.001	0.031	0.031*
Mothers’ modeling → Adolescents’ AHEI → Fathers’ SWFoL	0.008	−0.008	0.024	0.331
Fathers’ modeling → Mothers’ AHEI → Fathers’ SWFoL	0.006	−0.002	0.013	0.161
Fathers’ modeling → Fathers’ AHEI → Fathers’ SWFoL	0.034	0.016	0.053	<0.001***
Fathers’ modeling → Adolescents’ AHEI → Fathers’ SWFoL	0.004	−0.005	0.012	0.362
Mothers’ modeling → Mothers’ AHEI → Adolescents’ SWFoL	0.033	−0.001	0.066	0.059
Mothers’ modeling → Fathers’ AHEI → Adolescents’ SWFoL	−0.001	−0.010	−0.008	0.769
Mothers’ modeling → Adolescents’ AHEI → Adolescents’ SWFoL	0.010	−0.009	0.028	0.298
Fathers’ modeling → Mothers’ AHEI → Adolescents’ SWFoL	0.006	−0.005	0.015	0.165
Fathers’ modeling → Fathers’ AHEI → Adolescents’ SWFoL	−0.003	−0.022	0.016	0.769
Fathers’ modeling → Adolescents’ AHEI → Adolescents’ SWFoL	0.005	−0.005	0.015	0.327

*SWFoL, Satisfaction with food-related life.*

*p < 0.05, **p < 0.01, ***p < 0.001.

As we find neither direct nor indirect effects of parents’ modeling on the partners SWFoL, H7a is not supported. H7b is supported only for mothers. In addition, we find that the effect of parents’ modeling on their own SWFoL is partly mediated by their diet quality. These findings partially support the mediating role of diet quality between parents’ modeling and their own SWFoL, while they did not support the mediating role of the adolescent’s diet quality.

## Discussion

Focusing on different-sex dual-earner parents with adolescent children, this study tested the actor and partner effects between parents’ modeling and the three family members’ diet quality and SWFoL; and whether diet quality has a mediating role between both parents’ modeling and the three family members’ SWFoL. Using the APIM approach, our results showed that both mothers’ and fathers’ modeling can enhance their own and their adolescents’ diet quality, whereas one parent’s modeling also improves the other parent’s diet quality, regardless of the parents’ gender. Modeling and higher diet quality are directly associated with higher SWFoL in both parents. However, different patterns emerged in the direct and indirect links between modeling, diet quality and SWFoL according to the parent’s gender. Findings are discussed in detail below by examining actor and partner effects, and the mediating role of diet quality.

### Actor Effects

A positive relationship was found between each parent’s modeling of healthy food choices and their own diet quality (H1). These results provide support to the findings reported by Fleary and Ettiene (29) showing that parents’ FPP are indicative of their own dietary behavior, focusing on fruit and vegetable consumption in a sample of parent-adolescent dyads in the US. Our results expand on this knowledge by showing that parental modeling is another structured FPP that is indicative of parents’ dietary behavior. It should be noted, however, that these results are framed within the first months of the COVID-19 pandemic. The frequency of family meals reported in this study was higher than the one observed in studies conducted in Chile before the pandemic (36, 39), while the frequency of homemade meals was high. Therefore, the positive relationship between both parents’ modeling and their own diet may be related to the higher frequency of family meals during lockdown, which have been associated with healthier eating behaviors in different countries (11, 12, 14).

The second hypothesis testing actor effects stated that diet quality is positively associated with SWFoL for fathers (H3a) mothers (H3b), and adolescents (H3c). This hypothesis was supported for mothers and fathers, supporting previous studies showing that healthier eating habits are positively associated with higher levels of SWFoL in adult samples [e.g., (36, 39, 54–57)]. However, the lack of association between adolescents’ healthier diet and SWFoL contradicts previous studies in adolescent samples from different countries (39, 57, 58). Possible explanations to this latter finding may be related to shifting food preferences during the adolescence, or to changes due to the pandemic. The search for independence in adolescents also occurs in the food domain, as they increasingly choose what to eat and where, when and with whom (5, 6, 9). Research has also shown that adolescents prioritize hedonic food consumption over a healthy and nutritious consumption, and that they associate eating unhealthy/tastier food with higher SWFoL (59). Moreover, during the pandemic, adolescents have remained closer to their parents and away from their peers (73), so their diet might have depended more on what they are served (i.e., healthier food) rather than on what they want (i.e., enjoyable food).

Lastly, actor effects showed that modeling of healthy food choices is positively associated with SWFoL, for mothers and fathers (H6). Hence, part of this effect is observed because modeling is reflective of parents’ own eating behaviors, and these eating behaviors, in turn, result in higher levels of SWFoL (36, 39, 54–57). The direct, unmediated effect of both parents’ modeling on their own SWFoL may be related to the possibility of improving their children’s diet quality and wellbeing (2, 25). Overall, these findings are notable because they indicate that another structured FPP (i.e., parental modeling) directly and positively influences parents’ SWFoL. This result contributes to knowledge regarding FPP and their potential link to higher levels of SWFoL in parents (4, 39, 41–43).

Our results also show that both mothers’ and fathers’ SWFoL is positively influenced by exerting a positive FPP. The association for fathers was of medium strength, while the same association in mothers was of low strength. Although further research is needed regarding the association between fathers’ FPP and their own SWFoL, it is feasible that this result may be reflecting a higher concern of fathers in engaging in healthy eating behaviors during the pandemic, as it has been reported in different countries (13, 32). However, it is also possible that fathers’ modeling increased during the pandemic in keeping with a higher frequency of family meals in comparison to the pre-pandemic period (36, 39). A higher frequency of family meals has been also associated with healthier diets (11, 12, 14) and with higher emotional wellbeing during the pandemic in young adults from the US (12). Thus, future research is needed to corroborate if the medium strength association between fathers’ modeling and their own SWFoL remains beyond the pandemic or whether it is a consequence of the pandemic.

### Partner Effects

The first hypothesis testing partner effects stated that modeling of healthy food choices of one parent is positively associated with the diet quality of the other parent (H2a) and of the adolescent (H2b). Hypothesis 2a was supported for both parents, as mothers’ modeling positively influenced the fathers’ diet quality, and vice versa. These results show that modeling exerted by one parent to enhance children’s diet quality also positively influences the other parent’s diet quality, regardless of the parent’s gender (50, 53). However, our results contradict previous studies reporting that women are more influenced by their partner than men (51, 52), as the association between mothers’ modeling and the fathers’ diet quality was of similar strength in comparison with the association between fathers’ modeling and the mothers’ diet quality. Therefore, both parents’ modeling is important to enhance the diet quality of both members of the couple.

Mothers’ and fathers’ modeling was also positively related to their adolescent children’s diet quality (H2b). This result supports previous studies reporting that modeling is associated with an improvement in the adolescents’ diet quality, a higher consumption of healthy foods, and with lower consumption of unhealthy foods, mainly in samples of mother-adolescent dyads in developed countries (8, 22, 24, 48, 49). Our results also provide support to the scarce evidence showing that fathers’ modeling is related to positive dietary outcomes in adolescents (27, 34). Furthermore, the positive association between both parents’ modeling and their adolescent children’s diet quality also provides support to the findings reported by Jaeger et al. (21), who found that both parents reported similar levels of modeling to promote healthier eating habits (fruit and vegetable consumption) in their adolescent children. Thus, although research have highlighted the important role that mothers play in explaining their children’s food intake (20, 28, 33, 34, 41), our results support that both parents have a role in modeling healthy food-related behaviors among youth (27). The fathers’ modeling influence on their adolescent children’s diet quality may indicate an increased involvement of fathers in their children’s eating habits (19, 28, 30, 31), linked to societal changes in terms of gender roles, or it may be associated with the pandemic. The latter option, an increase in the fathers’ involvement in food-related tasks, has been consistently reported in the early stages of this health crisis (13, 32). Therefore, as it was previously posed, future research should assess if the positive association between fathers’ modeling and diet quality remains after the pandemic.

Actor effects were of medium strength (i.e., the influence of each parent’s modeling on their own diet quality), while partner effects were of low strength (i.e., the influence of one parent’s modeling on the other parent’s diet quality, as well as the influence of each parent’s modeling on their adolescent children’s diet quality). Although previous research using the APIM approach to study reciprocal influences among members of a dyad or between family members have reported similar results (32, 74), this finding is notable considering that FPP are used by parents to influence their children’s eating habits. By contrast, our results suggest that parent’s modeling have a stronger influence on their own diet quality than on their adolescent children’s diet quality.

The second hypothesis testing partner effects posed that diet quality of one parent is positively associated with SWFoL of the other parent (H4a) and of the adolescents (H4b). Hypothesis 4a was partially supported, as the mothers’ diet quality positively influenced the fathers’ SWFoL, but not vice versa, while hypothesis 4b was not supported. A possible explanation for this result may be related to the lower AHEI scores (indicating diet quality) in fathers. Although all three family members had AHEI scores in the range that their diet quality “requires changes,” fathers’ diet quality is the worst among the three family members under study. Therefore, it can be hypothesized that the improvement of the fathers’ diet quality associated with their own modeling is not enough to positively influence the mothers’ SWFoL, while a similar explanation may be true in the opposite direction, meaning that mothers’ diet quality positively influences fathers’ SWFoL because mothers have a higher diet quality than fathers. Regarding hypothesis 4a, the lack of significant associations between both parents’ diet quality and their adolescent children’s SWFoL is probably associated with their increased autonomy in food choices during adolescence (5, 6, 9). However, as the adolescent children’s SWFoL was also not influenced by their own diet quality (actor effects), the probability of the existence of partner effects is almost inexistent (74).

Hypothesis 5 also tested partner effects but in the opposite direction, stating that diet quality of adolescents is positively associated with their parents’ SWFoL. This hypothesis was supported only for mothers. The results suggest that traditional gender-based demands and expectations remain, particularly in the Latin American context, where feeding children is still predominantly female labor even if mothers and other female caretakers have paid employment (20, 28, 33, 34, 41). It thus may be the case for these women that if they can perform as well at work than as in food-related tasks, including the promotion of healthy eating habits in their adolescent children, their self-confidence may be reinforced, positively influencing their SWFoL (75).

Hypothesis 7 stated that modeling of healthy food choices of one parent is positively associated with the SWFoL of the other parent (H7a) and of the adolescent (H7b). Whereas hypothesis 7a was not supported for mothers and fathers, hypothesis 7b was partially supported. Only the mothers’ modeling was related to their adolescent children’s SWFoL, and this relationship was not mediated by the adolescent’s quality of diet. Previous evidence on crossover effects involving SWFoL is mixed, with lack of significant partner effects in some studies (75), others reporting at least an asymmetrical partner effect [from women to men, (61)], and symmetrical partner effects [from women to men, and from men to women, (76)] in dual-earner couples. Therefore, it is possible to suggest that crossover relationships involving SWFoL are associated with the variables under study, as it has been reported by Yucel and Latshaw (77) regarding crossover effects in the work-family interface among couples. Nevertheless, it is also possible that the relationship between both parents’ modeling and SWFoL may occur by underlying mechanisms, as discussed below.

Regarding the positive crossover association between mothers’ modeling and their adolescent children’s SWFoL, one possible explanation for these results may be associated with the different socialization practices and social roles for women and men. The positive association between mothers’ modeling and adolescents’ SWFoL may be due to adolescents’ perception that their mothers are fulfilling their gender role, while the lack of relationship between fathers’ modeling and their adolescent children’s SWFoL may be reflecting that adolescents do not value the fathers’ modeling, because this task is not associated with the traditional men’s role within the family in the Latin American culture (4). Overall, our results show that FPP may not only positively influence the SWFoL of the parent who exert the FPP (39–41), but also that of their adolescent children through crossover.

#### Testing Mediating Roles of Diet Quality

The last hypothesis of this study (H8) tested the mediating role of the three family members’ diet quality between both parents’ modeling of healthy food choices and the three family members’ satisfaction with food related life (actor and partner effects). This hypothesis was partially supported for parents’ diet quality, and not supported for the adolescents’ diet quality. Diet quality shows an intra-individual mediating role between modeling and SWFoL in mothers and fathers. These findings show that modeling and SWFoL were also indirectly associated *via* diet quality regardless of the parent’s gender.

In addition, mothers’ modeling was indirectly associated with fathers’ SWFoL *via* fathers’ diet quality. This result underscores the important role that mothers have in modeling healthy eating behaviors in their family, positively influencing -directly or indirectly- their own, the fathers’, and their adolescent children’s diet quality and SWFoL. Although further research is needed to explain the lack of an inter-individual mediating role of mothers’ diet quality, this null finding may be related to the weak relationship between fathers’ modeling and mothers’ diet quality. A similar explanation may stand for the lack of a mediating role for the adolescents’ diet quality.

#### Limitations

This study is not without limitations. The first limitation is the cross-sectional design of this study, which does not allow to indicate causal relationships between the variables. A second limitation relates to the sample. Families were self-selected, and although they were representative of the Chilean population in terms of socioeconomic status (68), these families had more family members than the average Chilean family (78). Moreover, data were self-reported, and participants might have answered the questionnaires thinking about expectations regarding modeling practices, eating habits, and their overall food-related life. Another limitation pertains to the AHEI measure, which has been shown to be useful to evaluate diet quality, but it does not include all possible food groups nor the quantity consumed for each. Regarding the overall method, the design of this study predated the pandemic, and the questionnaire was not able to capture conditions specific to this ongoing event, such as the transition from commuting to working from home, or commuting during lockdown. Lastly, this study did not account for other FPP, the adolescent’s perception of parental modeling, nor restrictions related to the COVID-19 pandemic. Longitudinal designs, probabilistic sampling, and cross-cultural comparisons are needed in future studies to further explore the relationships seen in this study. In addition, research must include the perception of both parents and adolescents regarding FPP, and the family-level assessment of diverse FPP, such as other structured ones, and those included in the coercive control and autonomy support or promotion classification by Vaughn et al. (2).

## Conclusion and Implications

Despite these limitations, this is the first study that analyses actor and partner effects for the relationships between both parents’ modeling and the three family members’ diet quality and SWFoL in different-sex dual-earner parents with adolescent children. Results showed that one parent’s modeling enhanced their own, their adolescent children’s and the other parent’s diet quality, regardless of the parent’s gender. Both parents’ modeling was associated with their own SWFoL, directly and indirectly *via* their own diet quality. However, different gender patterns emerged among parents regarding one parent’s modeling and diet quality influence on the other parent’s and their adolescent children’s SWFoL. Only mothers’ modeling was positively associated with their adolescent children’s SWFoL; mothers’ diet quality was positively associated with fathers’ SWFoL; and mothers’ modeling was indirectly related to the father SWFoL, *via* the fathers’ diet quality.

These results have research implications. Research is needed based on possible reciprocal relationships between parents’ FPP and family members’ outcomes to assess the influence that parents’ FPP have on their own and their children’ diet quality and wellbeing. In addition, as FPP are also exerted by parents to prevent overweight and obesity in their children (1), future studies should also assess the influence that different FPP have on parents’ and children’s nutritional status at a family level. Furthermore, as different patterns emerged according to the parent’s gender, future research should corroborate if these differences are associated with the COVID-19 pandemic or whether they may persist post-pandemic. In addition, future research should explore possible moderators of the associations found in this study. For instance, the frequency of family meals, the family SES, and the mothers’ type of employment, due as control variables significantly affect diet quality or SWFoL.

Our results also entail practical implications. As the three family members have diets that require changes to be considered healthy, interventions should be targeted at a family level. For instance, both parents should be encouraged to have a healthy diet to be a model for their partner’s and adolescent children’s eating habits, and should be encouraged to use other positive FPP. Interventions and policies that foster motivation, knowledge, and access to resources to establish healthy diets is particularly relevant in dual-earner families with adolescent children, because workers’ high job demands can entail lower diet quality for the worker and their families (20, 45, 46), and because adolescents’ diet quality tends to decrease during this stage of life (7, 9). Furthermore, unhealthy diet has been associated with modern health-related issues such as obesity, diabetes, and cardiovascular disease, as well as with lower work performance in workers (79). Therefore, our findings also underscore the need for policymakers and organizations to promote healthy eating habits in working parents, with emphasis on working fathers.

## Data Availability Statement

The raw data supporting the conclusions of this article will be made available by the authors, without undue reservation.

## Ethics Statement

The studies involving human participants were reviewed and approved by the Ethics Scientific Committee of the Universidad de La Frontera. Written informed consent to participate in this study was provided by the participants’ legal guardian/next of kin.

## Author Contributions

BS conceptualized the study. EM-Z conducted the analysis. EM-Z and HP handled the databases. KB collected data. MS, GL, CA-B, and ML provided support throughout data collection and manuscript preparation. BS and LO wrote the first draft and the final version of the manuscript. KG provided support for manuscript preparation. All authors reviewed and approved the final version of the manuscript.

## Conflict of Interest

The authors declare that the research was conducted in the absence of any commercial or financial relationships that could be construed as a potential conflict of interest.

## Publisher’s Note

All claims expressed in this article are solely those of the authors and do not necessarily represent those of their affiliated organizations, or those of the publisher, the editors and the reviewers. Any product that may be evaluated in this article, or claim that may be made by its manufacturer, is not guaranteed or endorsed by the publisher.

## References

[1] Vaughn AE Tabak RG Bryant MJ Ward DS. Measuring parent food practices: a systematic review of existing measures and examination of instruments. *Int J Behav Nutr Phys Act.* (2013) 10:1–27. 10.1186/1479-5868-10-61 23688157 PMC3681578

[2] Vaughn AE Ward DS Fisher JO Faith MS Hughes SO Kremers SP Fundamental constructs in food parenting practices: a content map to guide future research. *Nutr Rev.* (2016) 74:98–117. 10.1093/nutrit/nuv061 26724487 PMC4892304

[3] Piccoli ÂB Neiva-Silva L Mosmann CP Musher-Eizenman D Pellanda LC. Adolescents’ perception of parental feeding practices: adaptation and validation of the comprehensive feeding practices questionnaire for Brazilian adolescents-the CFPQ-teen. *PLoS One.* (2017) 12:e0187041. 10.1371/journal.pone.0187041 29145485 PMC5690605

[4] Schnettler B Rojas J Grunert KG Lobos G Miranda-Zapata E Lapo M Family and food variables that influence life satisfaction of mother-father-adolescent triads in a South American country. *Curr Psychol.* (2021) 40:3747–64. 10.1007/s12144-019-00328-4

[5] Loth KA MacLehose RF Larson N Berge JM Neumark-Sztainer D. Food availability, modeling and restriction: how are these different aspects of the family eating environment related to adolescent dietary intake? *Appetite.* (2016) 96:80–6. 10.1016/j.appet.2015.08.026 26327222 PMC4684786

[6] Melbye EL Øgaard T Øverby NC. Validation of the comprehensive feeding practices questionnaire with parents of 10-to-12-year-olds. *BMC Med Res Methodol.* (2011) 11:113. 10.1186/1471-2288-11-113 21827703 PMC3175203

[7] Balantekin KN Anzman-Frasca S Francis LA Ventura AK Fisher JO Johnson SL. Positive parenting approaches and their association with child eating and weight: a narrative review from infancy to adolescence. *Pediatr Obes.* (2020) 15:e12722. 10.1111/ijpo.12722 32881344 PMC8018716

[8] Lopez NV Schembre S Belcher BR O’Connor S Maher JP Arbel R Parenting styles, food-related parenting practices, and children’s healthy eating: a mediation analysis to examine relationships between parenting and child diet. *Appetite.* (2018) 128:205–13. 10.1016/j.appet.2018.06.021 29920321 PMC7529118

[9] Gunther C Reicks M Banna J Suzuki A Topham G Richards R Food parenting practices that influence early adolescents’ food choices during independent eating occasions. *J Nutr Educ Behav.* (2019) 51:993–1002. 10.1016/j.jneb.2019.05.597 31221526

[10] Brizi A Biraglia A. “Do I have enough food?” How need for cognitive closure and gender impact stockpiling and food waste during the COVID-19 pandemic: a cross-national study in India and the United States of America. *Pers Individ Differ.* (2021) 168:110396. 10.1016/j.paid.2020.110396 32982000 PMC7501171

[11] Philippe K Chabanet C Issanchou S Monnery-Patris S. Child eating behaviors, parental feeding practices and food shopping motivations during the COVID-19 lockdown in France: (how) did they change? *Appetite.* (2021) 161:105132. 10.1016/j.appet.2021.105132 33493611 PMC7825985

[12] Berge JM Hazzard VM Larson N Hahn SL Emery RL Neumark-Sztainer D. Are there protective associations between family/shared meal routines during COVID-19 and dietary health and emotional well-being in diverse young adults? *Prev Med Rep.* (2021) 24:101575. 10.1016/j.pmedr.2021.101575 34631398 PMC8487301

[13] Grunert KG De Bauw M Dean M Lähteenmäki L Maison D Pennanen K No lockdown in the kitchen: how the COVID-19 pandemic has affected food-related behaviours. *Food Res Int.* (2021) 150:110752. 10.1016/j.foodres.2021.110752 34865770 PMC8520572

[14] Jansen E Thapaliya G Aghababian A Sadler J Smith K Carnell S. Parental stress, food parenting practices and child snack intake during the COVID-19 pandemic. *Appetite.* (2021) 161:105119. 10.1016/j.appet.2021 33450298 PMC7987761

[15] Schnettler B Miranda-Zapata E Orellana L Grunert KG Poblete H Lobos G Work-to-family enrichment and atmosphere of family meals influence satisfaction with food-related life: an actor-partner interdependence approach in dual-earner parents with adolescent children. *Food Qual Prefer.* (2022) 97:104471. 10.1016/j.foodqual.2021.104471

[16] Jennings KM Loth KA Tate AD Miner MH Berge JM. Application of latent profile analysis to define subgroups of parenting styles and food parenting practices. *Appetite.* (2019) 139:8–18. 10.1016/j.appet.2019.04.001 30965046 PMC6556132

[17] Patel C Karasouli E Shuttlewood E Meyer C. Food parenting practices among parents with overweight and obesity: a systematic review. *Nutrients.* (2018) 10:1966. 10.3390/nu10121966 30545102 PMC6316864

[18] Musher-Eizenman D Holub S. Comprehensive feeding practices questionnaire: validation of a new measure of parental feeding practices. *J Pediatr Psychol.* (2007) 32:960–72. 10.1093/jpepsy/jsm037 17535817

[19] Rahill S Kennedy A Kearney J. A review of the influence of fathers on children’s eating behaviours and dietary intake. *Appetite.* (2020) 147:104540. 10.1016/j.appet.2019.104540 31783065

[20] Garrido-Fernández A García-Padilla FM Sánchez-Ramos JL Gómez-Salgado J Sosa-Cordobés E. The family as an actor in high school students’ eating habits: a qualitative research study. *Foods.* (2020) 9:419. 10.3390/foods9040419 32260058 PMC7230543

[21] Jaeger MM Vieth G Rothman AJ Simpson JA. Parents’ use of intentional modeling and social control to influence their adolescent’s health behavior: findings from the FLASHE study. *J Soc Pers Relationsh.* (2021) 38:2722–41. 10.1177/02654075211020136

[22] Thomson JL Hennessy E Landry AS Goodman MH. Patterns of food parenting practices regarding junk food and sugary drinks among parent-child dyads. *Nutr J.* (2020) 19:91. 10.1186/s12937-020-00610-3 32847599 PMC7448982

[23] Yee AZ Lwin MO Ho SS. The influence of parental practices on child promotive and preventive food consumption behaviors: a systematic review and meta-analysis. *Int J Behav Nutr Phys Act.* (2017) 14:1–14. 10.1186/s12966-017-0501-3 28399881 PMC5387370

[24] Zarychta K Mullan B Luszczynska A. It doesn’t matter what they say, it matters how they behave: parental influences and changes in body mass among overweight and obese adolescents. *Appetite.* (2016) 96:47–55. 10.1016/j.appet.2015.08.040 26341954

[25] Beckers D Karssen LT Vink JM Burk WJ Larsen JK. Food parenting practices and children’s weight outcomes: a systematic review of prospective studies. *Appetite.* (2021) 158:105010. 10.1016/j.appet.2020.105010 33075443

[26] Blaine RE Kachurak A Davison KK Klabunde R Fisher JO. Food parenting and child snacking: a systematic review. *Int J Behav Nutr Phys Act.* (2017) 14:146. 10.1186/s12966-017-0593-9 29096640 PMC5668962

[27] Baltaci A Alvarez de Davila S Reyes Peralta AO Laska MN Larson N Hurtado GA Adolescent-reported Latino fathers’ food parenting practices and family meal frequency are associated with better adolescent dietary intake. *Int J Environ Res Public Health.* (2021) 18:8226. 10.3390/ijerph18158226 34360517 PMC8346089

[28] De-Jongh O Tugault-Lafleur CN O’Connor TM Hughes SO Mâsse LC. Are fathers’ and mothers’ food parenting practices differentially associated with children’s eating behaviors? *Appetite.* (2021) 166:105434. 10.1016/j.appet.2021.105434 34107293

[29] Fleary SA Ettienne R. The relationship between food parenting practices, parental diet and their adolescents’ diet. *Appetite.* (2019) 135:79–85. 10.1016/j.appet.2019.01.008 30639293

[30] Litchford A Roskos MRS Wengreen H. Influence of fathers on the feeding practices and behaviors of children: a systematic review. *Appetite.* (2020) 147:104558. 10.1016/j.appet.2019.104558 31870933

[31] Sharif MZ Alcalá HE Albert SL Fischer H. Deconstructing family meals: do family structure, gender and employment status influence the odds of having a family meal? *Appetite.* (2017) 114:187–93. 10.1016/j.appet.2017.03.032 28347778 PMC5926186

[32] Schnettler B Orellana L Miranda-Zapata E Saracostti M Poblete H Lobos G Diet quality during the COVID-19 pandemic: effects of workplace support for families and work-to-family enrichment in dual-earner parents with adolescent children. *Appetite.* (2022) 169:105853. 10.1016/j.appet.2021.105823 34822922 PMC8611499

[33] van den Broek N Larsen J Verhagen M Burk WJ Vink JM. Is adolescents’ food intake associated with exposure to the food intake of their mothers and best friends? *Nutrients.* (2020) 12:786. 10.3390/nu12030786 32192005 PMC7146583

[34] Zhang Y Reyes Peralta A Arellano Roldan Brazys P Hurtado GA Larson N Reicks M. Development of a survey to assess Latino fathers’ parenting practices regarding energy balance–related behaviors of early adolescents. *Health Educ Behav.* (2020) 47:123–33. 10.1177/1090198119878769 31597482

[35] Verjans-Janssen S Van Kann D Kremers S Vos S Jansen M Gerards S. A cross-sectional study on the relationship between the family nutrition climate and children’s nutrition behavior. *Nutrients.* (2019) 11:2344. 10.3390/nu11102344 31581699 PMC6836050

[36] Schnettler B Lobos G Miranda-Zapata E Denegri M Ares G Hueche C. Diet quality, satisfaction with life, family life and food-related life across families: a cross-sectional pilot study with mother-father-adolescent triads. *Int J Environ Res Public Health.* (2017) 14:1313. 10.3390/ijerph14111313 29109387 PMC5707952

[37] Kerr ME Bowen M. *Family Evaluation.* New York, NY: Norton (1988).

[38] Ministerio de Salud de Chile. *Encuesta Nacional de Consumo Alimentario.* (2021). Available online at: https://www.minsal.cl/sites/default/files/ENCA.pdf (accessed July 14, 2021).

[39] Schnettler B Grunert KG Lobos G Miranda-Zapata E Denegri M Hueche C. Maternal food-related practices, quality of diet, and well-being: profiles of Chilean mother-adolescent dyads. *J Nutr Educ Behav.* (2018) 50:776–87. 10.1016/j.jneb.2018.03.003 29625914

[40] Schnettler B Grunert KG Lobos G Miranda-Zapata E Denegri M Ares G A latent class analysis of family eating habits in families with adolescents. *Appetite.* (2018) 129:37–48. 10.1016/j.appet.2018.06.035 29966728

[41] Schnettler B Grunert KG Lobos G Miranda-Zapata E Denegri M Hueche C. Exploring relationships between family food behaviour and well-being in single-headed and dual-headed households with adolescent children. *Curr Psychol.* (2021) 40:585–600. 10.1007/s12144-018-9974-8

[42] Utter J Denny S Lucassen M Dyson B. Adolescent cooking abilities and behaviors: associations with nutrition and emotional well-being. *J Nutr Educ Behav.* (2016) 48:35–41. 10.1016/j.jneb.2015.08.016 26411900

[43] Utter J Denny S Farrant B Cribb S. Feasibility of a family meal intervention to address nutrition, emotional wellbeing, and food insecurity of families with adolescents. *J Nutr Educ Behav.* (2019) 51:885–92. 10.1016/j.jneb.2019.03.015 31005604

[44] Grunert KG Dean M Raats MM Nielsen NA Lumbers M. A measure of satisfaction with food-related life. *Appetite.* (2007) 49:486–93. 10.1016/j.appet.2007.03.010 17481776

[45] Djupegot IL Nenseth CB Bere E Bjørnarå HBT Helland SH Øverby NC The association between time scarcity, sociodemographic correlates and consumption of ultra-processed foods among parents in Norway: a cross-sectional study. *BMC Public Health.* (2017) 17:447. 10.1186/s12889-017-4408-3 28506318 PMC5433068

[46] Takeda W Melby MK Ishikawa Y. Who eats with family and how often? Household members and work styles influence frequency of family meals in urban Japan. *Appetite.* (2018) 125:160–71. 10.1016/j.appet.2016.04.034 29447994

[47] Kenny DA Kashy DA Cook WL. *Dyadic Data Analysis.* New York, NY: Guilford press (2006).

[48] Loth KA Friend S Horning ML Neumark-Sztainer D Fulkerson JA. Directive and non-directive food-related parenting practices: associations between an expanded conceptualization of food-related parenting practices and child dietary intake and weight outcomes. *Appetite.* (2016) 107:188–95. 10.1016/j.appet.2016.07.036 27486926 PMC5112107

[49] Ma Z Hample D. Modeling parental influence on teenagers’ food consumption: an analysis using the family life, activity, sun, health, and eating (FLASHE) survey. *J Nutr Educ Behav.* (2018) 50:1005–14. 10.1016/j.jneb.2018.07.005 30414664

[50] Côté M Gagnon-Girouard MP Sabourin S Bégin C. Emotion suppression and food intake in the context of a couple discussion: a dyadic analysis. *Appetite.* (2018) 120:109–14. 10.1016/j.appet.2017.08.029 28864258

[51] Schnettler B Miranda-Zapata E Orellana L Bech-Larsen T Grunert KG. The effects of actor-partner’s meal production focus on satisfaction with food related life in cohabiting couples. *Food Qual Prefer.* (2020) 84:103949. 10.1016/j.foodqual.2020.103949

[52] van Vleet M Helgeson V Korytkowski M Seltman H Hausmann L. Communally coping with diabetes: an observational investigation using the actor-partner interdependence model. *J Fam Psychol.* (2018) 32:654–63. 10.1037/fam0000414 29809019 PMC6082133

[53] Cornelius T Desrosiers A Kershaw T. Spread of health behaviors in young couples: how relationship power shapes relational influence. *Soc Sci Med.* (2016) 165:46–55. 10.1016/j.socscimed.2016.07.030 27494239 PMC5003715

[54] Ismael D Ploeger A. The potential influence of organic food consumption and intention-behavior gap on consumers’ subjective wellbeing. *Foods.* (2020) 9:650. 10.3390/foods9050650 32443595 PMC7278807

[55] Liu R Grunert KG. Satisfaction with food-related life and beliefs about food health, safety, freshness and taste among the elderly in China: a segmentation analysis. *Food Qual Prefer.* (2019) 79:103775. 10.1016/j.foodqual.2019.103775

[56] Oliveira L Poínhos R de Almeida MDV. Food-related quality of life among older adults living in community: a multi-factorial approach. *Clin Nutr ESPEN.* (2021) 44:224–9. 10.1016/j.clnesp.2021.06.013 34330470

[57] Schnettler B Grunert KG Lobos G Miranda-Zapata E Denegri M Lapo M Maternal well-being, food involvement and quality of diet: profiles of single mother-adolescent dyads. *Child Youth Serv Rev.* (2019) 96:336–45. 10.1016/j.childyouth.2018.11.020

[58] Vaqué-Crusellas C González M Casas F. Does satisfaction with food matter? testing the personal well-being index-school children (PWI-SC) with an additional item on satisfaction with food on a sample of 10 to 12-year-olds. *Child Ind Res.* (2015) 8:961–73. 10.1007/s12187-015-9301-y

[59] Schnettler B Hueche C Andrades J Ares G Miranda H Orellana L How is satisfaction with food-related life conceptualized? A comparison between parents and their adolescent children in dual-headed households. *Food Qual Prefer.* (2020) 86:104021. 10.1016/j.foodqual.2020.104021

[60] Diener ED Emmons RA Larsen RJ Griffin S. The satisfaction with life scale. *J Pers Assess.* (1985) 49:71–5. 10.1207/s15327752jpa4901_13 16367493

[61] Schnettler B Miranda-Zapata E Grunert KG Lobos G Lapo M Hueche C. Satisfaction with food-related life and life satisfaction: a triadic analysis in dual-earner parents families. *Cad Saude Publica.* (2020) 36:e00090619. 10.1590/0102-311X00090619 32267375

[62] O’Connor T Perez O Garcia IC Gallagher M. Engaging Latino fathers in children’s eating and other obesityrelated behaviors: a review. *Curr Nutr Rep.* (2018) 7:29–38. 10.1007/s13668-018-0225-2 29892790

[63] Melbye EL Hansen H. Promotion and prevention focused feeding strategies: exploring the effects on healthy and unhealthy child eating. *Biomed Res Int.* (2015) 2015:306306. 10.1155/2015/306306 26380269 PMC4561864

[64] Schnettler B Denegri M Miranda-Zapata E Saracostti M. *Fondecyt Project 1190017 “Interrelaciones Trabajo-Familia-Alimentación y Satisfacción Vital en Familias Nucleares con dos Ingresos Parentales e Hijos Adolescentes, en Tres Regiones de Chile: Un Estudio Transversal y Longitudinal.* Santiago: Conicyt (2018).

[65] Kennedy ET Ohls J Carlson S Fleming K. The healthy eating index: design and applications. *J Am Diet Assoc.* (1995) 95:1103–8. 10.1016/S0002-8223(95)00300-2 7560680

[66] Norte AI Ortiz R. Calidad de la dieta española según el índice de alimentación saludable. *Nutr Hosp.* (2011) 26:330–6. 10.3305/nh.2011.26.2.4630 21666971

[67] Schnettler B Miranda H Sepúlveda J Denegri M Mora M Lobos G Psychometric properties of the satisfaction with food-related life scale: application in southern Chile. *J Nutr Educ Behav.* (2013) 45:443–9. 10.1016/j.jneb.2012.08.003 23337474

[68] Asociación de Investigadores de Mercado. *Cómo Clasificar los Grupos Socioeconómicos en Chile.* (2016). Available online at: http://www.iab.cl/wp-content/uploads/2015/12/Presentaci%C3%B3n-final-AIM.pdf (accessed May 23, 2018).

[69] Hu LT Bentler PM. Cutoff criteria for fit indexes in covariance structure analysis: conventional criteria versus new alternatives. *Struct Equ Model.* (1999) 6:1–55. 10.1080/10705519909540118

[70] Lau RS Cheung GW. Estimating and comparing specific mediation effects in complex latent variable models. *Organ Res Methods.* (2012) 15:3–16. 10.1177/1094428110391673

[71] World Health Organization [WHO]. *Child Growth and Development.* (2007). Available online at: http://www.who.int/childgrowth/en/ (accessed May 26, 2016).

[72] Ministerio de Salud de Chile. *Patrones de Crecimiento Para la Evaluación Nutricional de Niños, Niñas y Adolescentes, Desde el Nacimiento Hasta los 19 Años de Edad.* (2018). Available online at: http://www.bibliotecaminsal.cl/wp/wp-content/uploads/2018/03/2018.03.16-Patrones-de-crecimiento-para-la-evaluaci%C3%B3n-nutricional-de-ni%C3%B1os-ni%C3%B1as-y-adolescentes-2018.pdf (accessed May 26, 2021).

[73] Magson N Freeman J Rapee R Richardson C Oar E Fardouly J. Risk and protective factors for prospective changes in adolescent mental health during the COVID-19 pandemic. *J Youth Adolesc.* (2020) 50:44–57. 10.1007/s10964-020-01332-9 33108542 PMC7590912

[74] Garcia RL Kenny DA Ledermann T. Moderation in the actor–partner interdependence model. *Pers Relationsh.* (2015) 22:8–29. 10.1111/pere.12060

[75] Schnettler B Miranda-Zapata E Orellana L Poblete H Lobos G Lapo M Domain satisfaction and overall life satisfaction: testing the spillover-crossover model in Chilean dual-earner couples. *Int J Environ Res Public Health.* (2020) 17:7554. 10.3390/ijerph17207554 33080810 PMC7589047

[76] Schnettler B Miranda-Zapata E Grunert KG Lobos G Lapo M Hueche C. Testing the spillover-crossover model between work-life balance and satisfaction in different domains of life in dual-earner households. *Appl Res Qual Life.* (2021) 16:1475–501. 10.1007/s11482-020-09828-z

[77] Yucel D Latshaw BA. Spillover and crossover effects of work-family conflict among married and cohabiting couples. *Soc Ment Health.* (2020) 10:35–60. 10.1177/2156869318813006

[78] Instituto Nacional de Estadísticas. *Censo de Población y Vivienda 2017.* (2018). Available online at: http://www.censo2017.cl/ (accessed August 5, 2019).

[79] Liu Y Song Y Koopmann J Wang M Chang CHD Shi J. Eating your feelings? Testing a model of employees’ work-related stressors, sleep quality, and unhealthy eating. *J Appl Psychol.* (2017) 102:1237. 10.1037/apl0000209 28394149

