# Supplementary material for: Parents’ Modeling During the COVID-19 Pandemic: Influences on Family Members’ Diet Quality and Satisfaction With-Food-Related Life in Dual-Earner Parents With Adolescent Children
Source: Front Nutr. 2022 May 18;9:902103. doi: 10.3389/fnut.2022.902103 (PMC9158745; doi:10.3389/fnut.2022.902103)
Supplement: Supplementary file 1 [file Table_1.DOCX]

**Supplementary information.**

Standardized effects estimate of control variables on diet quality (measured by the Adapted Healthy Eating Index, AHEI) and Satisfaction with Food-related life (SWFoL) in dual-earner parents with adolescent children.

|  | Estimate | p-value |
| --- | --- | --- |
| Family socioeconomic status → Mothers’ AHEI | .170 | .001 ** |
| Number of children → Mothers’ AHEI | .027 | .629 |
| Mothers’ age → Mothers’ AHEI | .119 | .106 |
| Fathers’ age → Mothers’ AHEI | -.030 | .683 |
| Adolescents’ age → Mothers’ AHEI | -.036 | .443 |
| Mothers’ working hours → Mothers’ AHEI | -.004 | .944 |
| Fathers’ working hours → Mothers’ AHEI | -.051 | .317 |
| Mothers’ type of employment → Mothers’ AHEI | .116 | .035* |
| Fathers’ type of employment → Mothers’ AHEI | -.024 | .654 |
| Number of supper times per week eaten together → Mothers’ AHEI | .053 | .362 |
| Family socioeconomic status → Fathers’ AHEI | .042 | .401 |
| Number of children → Fathers’ AHEI | -.089 | .108 |
| Mothers’ age → Fathers’ AHEI | .012 | .863 |
| Fathers’ age → Fathers’ AHEI | .139 | .050 |
| Adolescents’ age → Fathers’ AHEI | .003 | .954 |
| Mothers’ working hours → Fathers’ AHEI | .031 | .566 |
| Fathers’ working hours → Fathers’ AHEI | .050 | .329 |
| Mothers’ type of employment → Fathers’ AHEI | .068 | .226 |
| Fathers’ type of employment → Fathers’ AHEI | .012 | .805 |
| Number of supper times per week eaten together → Fathers’ AHEI | .112 | .051 |
| Family socioeconomic status → Adolescents’ AHEI | .128 | .014 * |
| Number of children → Adolescents’ AHEI | -.057 | .267 |
| Mothers’ age → Adolescents’ AHEI | .129 | .082 |
| Fathers’ age → Adolescents’ AHEI | -.013 | .855 |
| Adolescents’ age → Adolescents’ AHEI | .081 | .111 |
| Mothers’ working hours → Adolescents’ AHEI | -.015 | .797 |
| Fathers’ working hours → Adolescents’ AHEI | -.030 | .551 |
| Mothers’ type of employment → Adolescents’ AHEI | .118 | .037 * |
| Fathers’ type of employment → Adolescents’ AHEI | .022 | .679 |
| Number of supper times per week eaten together → Adolescents’ AHEI | .106 | .037 * |
| Family socioeconomic status → Mothers’ SWFoL | .134 | .007 ** |
| Number of children → Mothers’ SWFoL | .056 | .293 |
| Mothers’ age → Mothers’ SWFoL | -.054 | .480 |
| Fathers’ age → Mothers’ SWFoL | .096 | .219 |
| Adolescents’ age → Mothers’ SWFoL | .023 | .653 |
| Mothers’ working hours → Mothers’ SWFoL | .038 | .493 |
| Fathers’ working hours → Mothers’ SWFoL | -.069 | .169 |
| Mothers’ type of employment → Mothers’ SWFoL | .077 | .141 |
| Fathers’ type of employment → Mothers’ SWFoL | .028 | .572 |
| Number of supper times per week eaten together → Mothers’ SWFoL | .053 | .289 |
| Family socioeconomic status → Fathers’ SWFoL | -.013 | .828 |
| Number of children → Fathers’ SWFoL | -051 | .348 |
| Mothers’ age → Fathers’ SWFoL | .027 | .734 |
| Fathers’ age → Fathers’ SWFoL | -.107 | .154 |
| Adolescents’ age → Fathers’ SWFoL | .020 | .710 |
| Mothers’ working hours → Fathers’ SWFoL | .061 | .266 |
| Fathers’ working hours → Fathers’ SWFoL | -.054 | .290 |
| Mothers’ type of employment → Fathers’ SWFoL | -.021 | .687 |
| Fathers’ type of employment → Fathers’ SWFoL | .066 | .187 |
| Number of supper times per week eaten together → Fathers’ SWFoL | .110 | .038 * |
| Family socioeconomic status → Adolescents’ SWFoL | -.003 | .953 |
| Number of children → Adolescents’ SWFoL | .036 | .504 |
| Mothers’ age → Adolescents’ SWFoL | -.055 | .464 |
| Fathers’ age → Adolescents’ SWFoL | -.022 | .767 |
| Adolescents’ age → Adolescents’ SWFoL | -.084 | .130 |
| Mothers’ working hours → Adolescents’ SWFoL | .041 | .461 |
| Fathers’ working hours → Adolescents’ SWFoL | -.063 | .225 |
| Mothers’ type of employment → Adolescents’ AHEI | .073 | .183 |
| Fathers’ type of employment → Adolescents’ SWFoL | .067 | .220 |
| Number of supper times per week eaten together → Adolescents’ SWFoL | .089 | .074 |

* p < .05

** p < .01

*** p < .001
